# Integrative molecular analysis of metastatic hepatocellular carcinoma

**DOI:** 10.1186/s12920-019-0586-4

**Published:** 2019-11-13

**Authors:** Dongfang Wang, Yanjing Zhu, Jing Tang, Qiuyu Lian, Guijuan Luo, Wen Wen, Michael Q. Zhang, Hongyang Wang, Lei Chen, Jin Gu

**Affiliations:** 1 MOE Key Laboratory of Bioinformatics, BNRIST Bioinformatics Division, Department of Automation, Tsinghua University, Beijing, 100084 China; 2 International Co-operation Laboratory on Signal Transduction, Eastern Hepatobiliary Surgery Institute, Second Military Medical University, Shanghai, 200438 China; 3 Department of Neurosurgery, Wuhan General Hospital, 627 Wuluo Road, Wuhan, 430070 China; 4 Department of Biological Sciences, Center for Systems Biology, University of Texas at Dallas, Richardson, TX 75080 USA

**Keywords:** Hepatocellular carcinoma, Integrative genomic analysis, Metastasis

## Abstract

**Background:**

Hepatocellular carcinoma (HCC) is the major type of primary liver cancer. Intrahepatic metastasis, such as portal vein tumor thrombosis (PVTT), strongly indicates poor prognosis of HCC. But now, there are limited understandings of the molecular features and mechanisms of those metastatic HCCs.

**Methods:**

To characterize the molecular alterations of the metastatic HCCs, we implemented an integrative analysis of the copy number variations (CNVs), DNA methylations and transcriptomes of matched adjacent normal, primary tumor and PVTT samples from 19 HCC patients.

**Results:**

CNV analysis identified a frequently amplified focal region chr11q13.3 and a novel deletion peak chr19q13.41 containing three miRNAs. The integrative analysis with RNA-seq data suggests that CNVs and differential promoter methylations regulate distinct oncogenic processes. Then, we used individualized differential analysis to identify the differentially expressed genes between matched primary tumor and PVTT of each patient. Results show that 5 out of 19 studied patients acquire evidential progressive alterations of gene expressions (more than 1000 differentially expressed genes were identified in each patient). While, another subset of eight patients have nearly identical gene expressions between the corresponding matched primary tumor and PVTT. Twenty genes were found to be recurrently and progressively differentially expressed in multiple patients. These genes are mainly associated with focal adhesion, xenobiotics metabolism by cytochrome P450 and amino acid metabolism. For several differentially expressed genes in metabolic pathways, their expressions are significantly associated with overall survivals and vascular invasions of HCC patients. The following transwell assay experiments validate that they can regulate invasive phenotypes of HCC cells.

**Conclusions:**

The metastatic HCCs with PVTTs have significant molecular alterations comparing with adjacent normal tissues. The recurrent alteration patterns are similar to several previously published general HCC cohorts, but usually with higher severity. By an individualized differential analysis strategy, the progressively differentially expressed genes between the primary tumor and PVTT were identified for each patient. A few patients aquire evidential progressive alterations of gene expressions. And, experiments show that several recurrently differentially expressed genes can strongly regulate HCC cell invasions.

## Background

Hepatocellular carcinoma (HCC) is one of the most common cancer types worldwide. More than 700,000 people were diagnosed yearly [1]. Of them, intrahepatic metastasis, such as portal vein tumor thrombosis (PVTT), is a strong indication of poor prognosis [2]. Characterizing the molecular alterations of the metastatic HCCs with PVTTs is important for understanding the molecular mechanisms during HCC progression and metastasis. Previous studies mainly focused on single molecular layer, such as somatic mutations [3], gene expressions [4, 5] or miRNA expressions [6]. Integrative analysis of multiple molecular levels can overcome the potential bias of any single level information and provide a broader understanding of the molecular subtyping and the driving molecular alterations of cancer [7–10]. Several recent integrative molecular projects of HCC rendered more novel systems biological insights than the previous single level studies [11–14]. In this study, we systematically examined the copy number variations (CNVs), DNA methylations, and transcriptomes of matched adjacent normal tissues, primary tumors, and portal vein tumor thrombi (PVTTs) from 19 HCC patients. Based on the integrative molecular profiles, we identified a set of recurrent CNVs, abnormal DNA methylations, and candidate drivers of the metastatic HCCs. We observed that most arm-level CNVs and focal amplified regions are consistent with the previous cohorts, but several focal regions (such as chr11q13.3) are much prevalent in our metastatic cohort.

Another important question is whether there exist progressive molecular alterations between primary tumors and matched PVTTs. Ye et al. found that the gene expression patterns of metastatic lesions are nearly identical to their corresponding primary HCCs [4]. Similar results are observed for somatic mutations and miRNA expressions: Huang et al. found that more than 94% somatic mutations are shared by primary tumor and PVTTs [3], and Wong et al. reported that no obvious differences of miRNA expressions could be found between primary HCCs and the venous metastases [6]. As these previous studies, computational analysis shows that the inter-patient differences are much larger than the intra-patient heterogeneities between matched primary tumor and PVTT in most cases. Few consistent molecular alterations can be found between primary tumors and matched PVTTs. However, we observed that a few patients may have progressive molecular alterations according to the clustering analysis. So, we used a novel individualized differential analysis strategy to identify the progressively differentially expressed genes between matched primary tumor and PVTT for each patient. Results show that different patients have very different numbers of progressively differentially expressed genes and five patients even have more than 1000 differentially expressed genes. Twenty genes, mainly associated with focal adhesion, xenobiotics metabolism by cytochrome P450, and amino acid metabolism, are found to be recurrently differentially expressed in multiple patients. The following validation experiments suggest that these genes can regulate invasive phenotypes of liver-derived cell lines.

## Methods

### Clinical samples

All samples used in this study were obtained from patients undergoing surgery for HCC at the Eastern Hepatobiliary Surgery Hospital (Shanghai, China). Patient samples were obtained following informed consent according to an established protocol approved by the Ethics Committee of Eastern Hepatobiliary Surgery Hospital. Frozen adjacent normal tissues, primary tumors, and PVTTs were derived from 19 HCC patients (median age 49, 17 male, 18 HBV positive, and no HCV infection detected). The majority of the primary tumors are larger than 5 cm (15 patients) and Edmondson-Steiner histological grades are III or IV. Please see detailed information in Additional file 1: Table S1.

### Total RNA preparation

Samples were treated with 1 mL TRIzol reagent (Life Technologies Cat.#15,596–026) according to manufacturer’s instructions. Nanodrop ND-1000 was used for RNA density/purity detection. Agilent BioAnalyzer 2100 was used for RNA quality control.

### Genomic DNA extraction

DNeasy Blood & Tissue Kit (QIAGEN Cat.#69,504) and RNase A (QIAGEN Cat.#19,101) were used for genomic DNA extraction according to manufacturer’s instructions.

### CNV analysis

Affymetrix CytoScan HD was used for CNV analysis. Raw CEL files were processed as segmentations files by Nexus Copy Number v7.5 (BioDiscovery) with default settings. Then, the segmentation files were used as inputs to GISTIC2 [15] with default parameters for analyzing arm-level and focal CNVs. To identify the significant variations, for arm-level CNVs, the cutoffs were set as frequency ≥ 0.5 and z-score ≥ 1.5. For focal CNVs, we used the default cutoffs as q-value < 0.05.

### DNA methylation analysis

Illumina HumanMethylation450 BeadChip was used to profile ~ 480,000 CpG methylation levels. Genome Studio was used to process .idat raw data into beta-values. The data points with *p*-value > 0.05 were treated as outliers. FastDMA [16] was used to identify differentially methylated sites and regions. A promoter region (from upstream 1500 bp to downstream 500 bp around the transcription start site) was identified as “hyper-methylated” with q-value <1e-4 and differential methylation level (tumor minus adjacent beta-value) > 0.2 for at least two promoter probes.

### miRNA-seq analysis

Total RNA was treated mirVanaTM miRNA Isolation Kit (Life Technologies Cat.#AM1560). 50 bp single-end sequencing was performed on Illumina HiSeq2500 platform. Raw reads were subjected to initial quality control using FastQC. miRDeep2 [17] was used to remove adapters and quantify miRNA expressions based on miRBase annotations (release 20) [18].

### RNA-seq analysis

rRNA depletion was conducted before RNA-seq library preparation using TruSeq Stranded Total RNA Library Prep Kit (Illumina Cat.#RS-122-2302). 100 bp paired-end sequencing was performed on Illumina HiSeq2500 platform. Raw reads were subjected to initial quality control using FastQC. TopHat [19] and Subread [20] were used for reads mapping and counting. EdgeR [21] was used to identify differentially expressed genes (paired test q-value < 0.01).

### Integrative analysis with RNA-seq

Non-parameter Spearman’s correlation was used to identify candidate genetic and epigenetic candidate driver genes. In this study, the genes expressions and CNV correlation > 0.4 or promoter DNA methylation correlation < − 0.4 are used as the cutoffs to select candidate drivers.

### Clustering analysis

LRAcluster [22] was used to visualize multi-omics data in two-dimensional principal subspace. Discretized CNVs, beta-values of promoter DNA methylation probes, normalized counts of coding genes, and normalized counts of miRNAs were used for the multi-omics integrative analysis. R package pvclust [23] was used for the hierarchical clustering.

### Individualized differential analysis for sequencing data (IDASeq)

IDASeq was developed for identifying individualized differentially expressed genes (or lncRNAs) using paired samples of each patient (see details in Additional file 2: Methods). The expression data of adjacent normal tissues were pooled to estimate the variations conditional on different expression means *σ*^2^ ∣ *μ*. The difference and mean of *i*-th gene of *j*-th patient’s paired primary tumor and PVTT samples were calculated as ${d}_{ij}={e}_{i,j}^p-{e}_{i,j}^t$ and ${\mu}_{ij}=\frac{e_{i,j}^p+{e}_{i,j}^t}{2}$, respectively. The statistical significance of the difference was calculated as *z*-score ${z}_{ij}=\frac{d_{ij}}{\sqrt{{\left.2{\sigma}^2\right|}_{\mu_{ij}}}}$ (*z*_*ij*_ follows standard normal distribution). The *p*-values, calculated from *z*-scores, were adjusted for each patient using BH correction. We set adjusted *p*-value < 0.1 to select differentially expressed genes for each patient. Then, a permutation test was used to empirically calculate the statistical significances of the recurrently differentially expressed genes.

### Third-party cohorts

Two cohorts, TCGA-LIHC (HCC samples only) and GSE54504, were used to compare the copy number analysis results. Two cohorts, TCGA-LIHC (HCC samples only) and GSE14520, were used for overall survival analysis. For a given gene, the samples were split into two groups according to its expression levels (above median and below median). Then, KM-test was used to compare the survivals between the two groups. Three cohorts, TCGA-LIHC (HCC samples only), GSE9843, and GSE19977 were used for vascular invasion analysis. The gene expressions were compared between the samples annotated with/without the vascular invasions in each dataset using Wilcox’s rank test.

### Cell cultures

Human HCC cell line HCC-LM3 (Cell Bank of Chinese Academy of Sciences (Shanghai), Cat. #TCHu94) and immortalized liver-derived cell line QSG-7701 (Cell Bank of Chinese Academy of Sciences (Shanghai), Cat. #GNHu7) were maintained in Dulbecco’s modified Eagle’s medium (DMEM; Gibco, USA) supplemented with 10% (v/v) fetal bovine serum (FBS). All cells were incubated at 37 °C in a humidified atmosphere of 5% CO_2_ (v/v) in air.

### Cell transfections

Human HCC-LM3 and QSG-7701 cells (5 × 10^5^ cells) were cultured in 6-well plates with antibiotics-free DMEM for 24 h and then subjected to transfection with siRNA using Lipofectamine™ 2000 (Invitrogen, USA) according to the manufacturer’s protocol. The sequences of the siRNAs and NC are provided in Additional file 3: Table S2.

### Transwell invasion assay

Cell invasion assay was performed in a 24-well transwell chamber (Corning, USA) with a pore size of 8 mm (Greiner Bio-One, USA). For migration assay, after the appropriate treatments, cells were trypsinized and seeded in the upper chamber at a density of 5 × 10^4^ cells/well in 300 μL of serum-free medium. Five hundred microliter of complete medium was added to the lower chamber as a chemo-attractant. After incubation for 24 h, the invaded cells were fixed with 4% paraformaldehyde, stained with 0.1% crystal violet, and quantified from microscopic fields.

## Results

### The recurrent genomic and epigenetic alterations

**Fig. 1:**
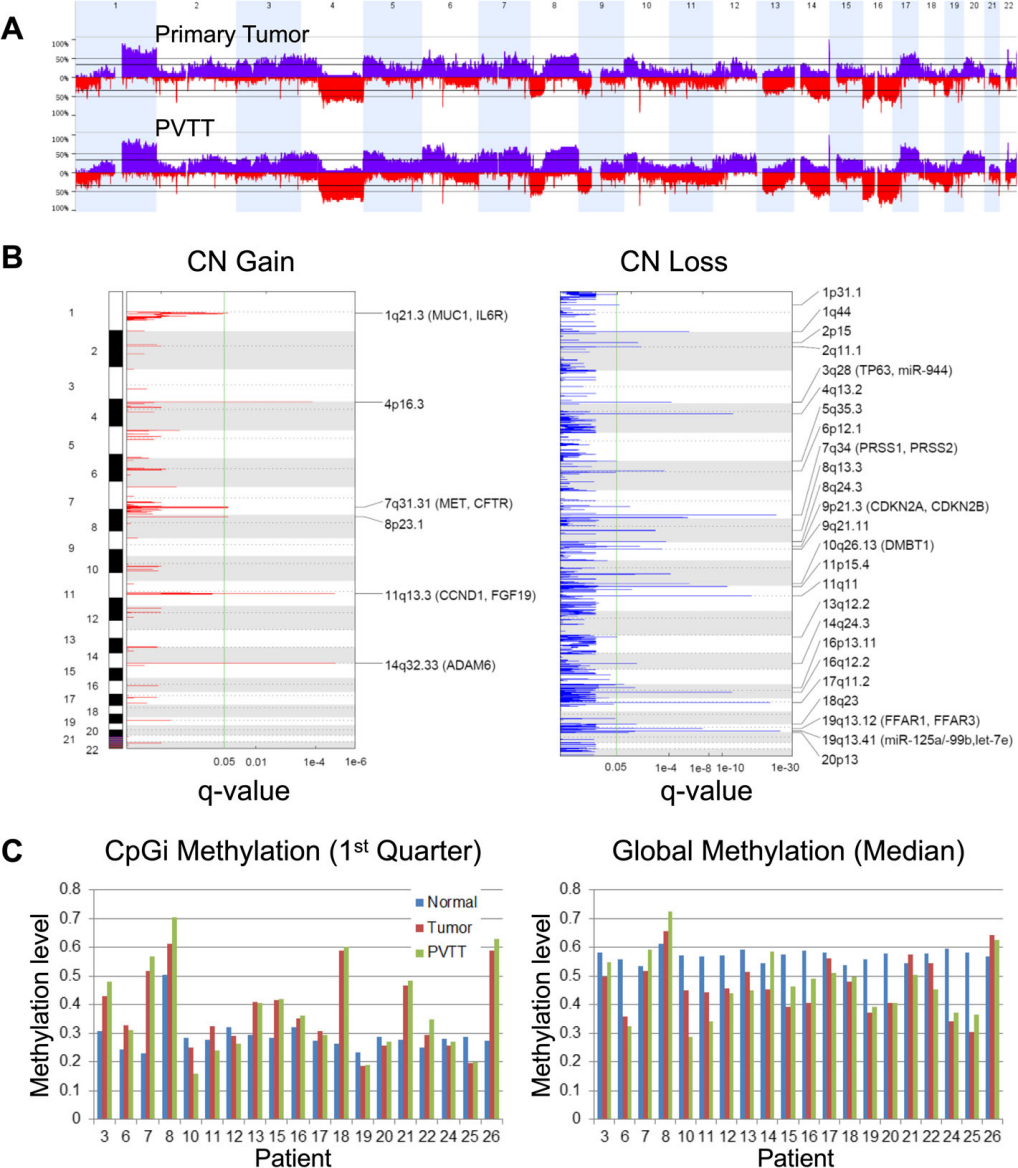
The genomic and epigenomic landscapes of primary hepatocellular carcinomas and PVTTs. **a** Copy number variations in primary tumor and PVTTs. **b** Focal copy number (CN) alterations detected by GISTIC2 (only the primary tumors were shown). **c** The DNA methylation levels in CpG islands and in whole genomes

CNV analysis indicates that the genomes of these metastatic HCCs (both primary tumors and PVTTs) are highly abnormal. The average percentage of genome affected by CNV is 31.2%. Recurrent arm-level copy number gains are found in 1q, 4p, 5p, 8q, 17q and loss in 4q, 8p, 9p, 11p/q, 13q, 14q, 16p/q, 17p, 19p (frequency ≥ 0.5 and z-score ≥ 1.5 by GISTIC2) (Fig. 1a), which are much more serious than previous studies (see the frequent arm-level CNVs reviewed by [24]). Six focal amplifications and 25 deletion regions were identified in primary tumors (Fig. 1b, Additional file 4: Table S3). The most significantly amplified region is located at 11q13.3 with 11 genes including *CCND1*, *FGF19*, *FGF3*, and *FGF4* (q-value 1.33e-05). Previous studies suggest that chr11q13.3 *CCND1*-*FGF19* focal amplification is strongly associated with HCC progression [25–27]. In our cohort, this region is amplified in 36.8% (7 out of 19) primary tumors. This ratio is much higher than another two previous studies (to make the results more comparable, we re-processed the raw CEL files with the same pipeline): 15.6% in GSE54504 (36 out of 231 samples, Fisher’s exact test *p*-value 0.042) and 14.7% in TCGA-LIHC (55 out of 375 samples, *p*-value 0.018). It suggests that *CCND1*-*FGF19* amplification is a candidate driver event for HCC metastases. For the copy number loss, a known deletion region 9p21.3 (q-value 5.85e-3) including *CDKN2A* and *CDKN2B*, was identified in our study. We also identified a novel deletion peak in 19q13.41 (q-value 6.57e-23, the second most significant peak) which contains three miRNAs, let-7e, miR-125a, and miR-99b. MiR-125a is a known tumor suppressor in HCC, which can inhibit cancer cell proliferation and metastasis [28]. Let-7e and miR-99b are also proved as tumor-suppressors in other solid tumors [29, 30]. The deletion of these miRNAs should play an important role in HCC progression.

Whole-genome DNA methylation analysis shows that about a half of tumors have global hypo-methylation patterns and ~ 40% tumors acquired strong CpG island methylator phenotype (CIMP) (Fig. 1c). A few gene promoters are strongly hyper-methylated. With a stringent threshold (q-value <1e-4 and differential methylation level > 0.2 for at least two promoter probes), 51 genes are hyper-methylated in their promoter regions (Additional file 5: Table S4). *NKX6–2*, *TBX15, CDKL2* are the top genes with ≥9 hyper-methylated probes in promoters. Three novel candidates with ≥6 hyper-methylated probes, *NKAPL*, *GRHL2*, and *EVX1*, were identified. Several other known promoter hyper-methylated genes were also confirmed in this study, such as *RASSF1*, *TSPYL5*, and *HOXA11* [13, 31, 32]. Interestingly, these hyper-methylated promoters are frequently associated with histone coding gene clusters (*HIST1H4F*, *HIST1H3G*, *HIST1H2BH*, and *HIST1H2BM*), but its functional consequence remains unclear.

### The integrative analysis with RNA-seq

**Fig. 2:**
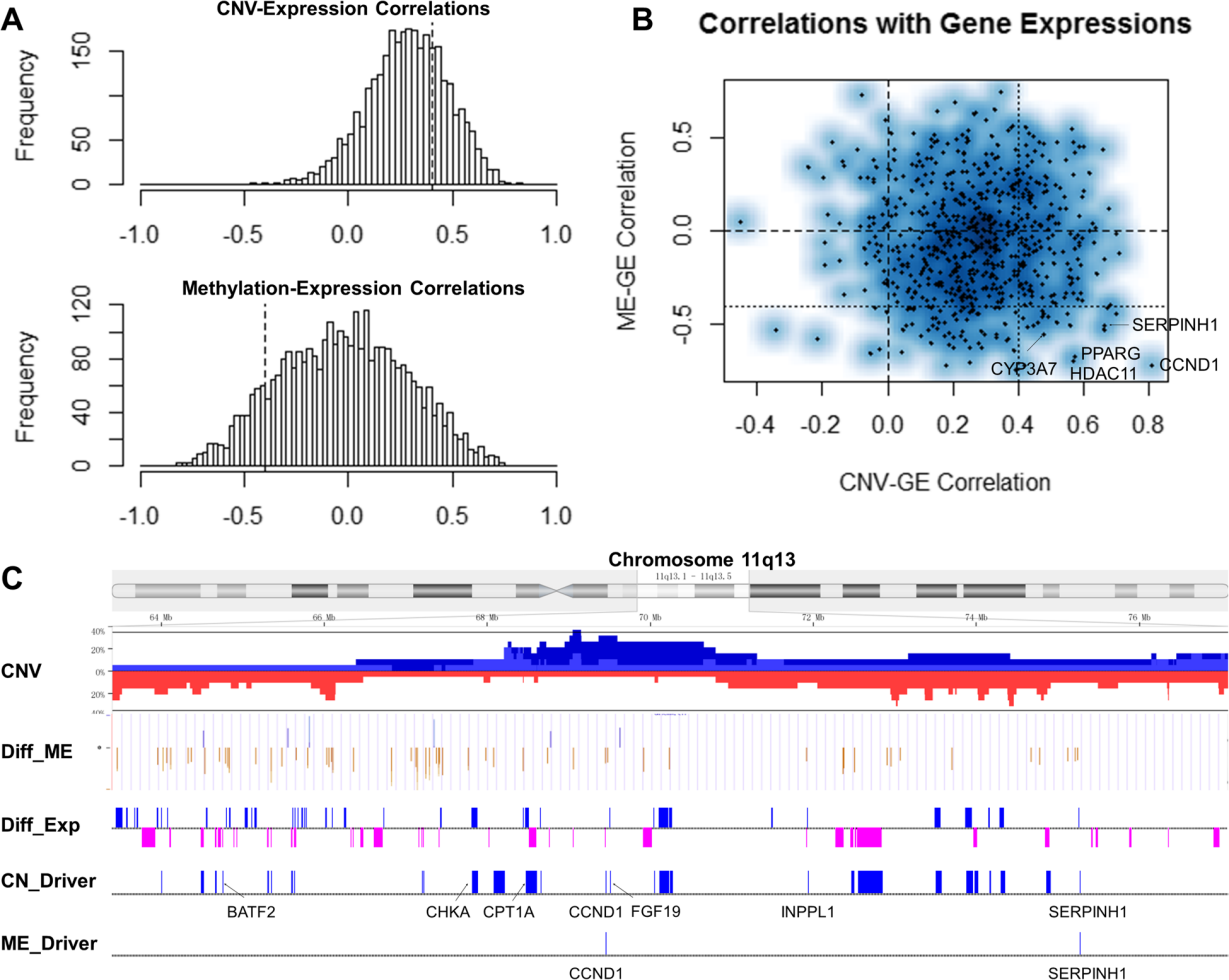
Candidate driver genes regulated by CNVs and promoter DNA methylations. **a** The correlations between gene expressions and CNVs/promoter DNA methylations. **b** Scatterplot of the correlations. A few top candidate drivers, whose expressions are significantly affected both by CNVs and promoter DNA methylations, are highlighted. **c** A hotspot genomic region of candidate driver genes (chromosome 11q13). The first row shows the accumulated CNVs, the second shows the differential DNA methylation levels in promoters, the third shows the differential gene expressions, the fourth indicates candidate genetic driver genes whose expressions are positively correlated with CNVs and the fifth indicates candidate epigenetic driver genes whose expressions are negatively correlated with promoter DNA methylations

About 7700 genes are significantly differentially expressed between primary tumors and adjacent normal tissues (EdgeR paired test q-value < 0.01) (Additional file 6: Table S5). Then, we combined CNVs and DNA methylations with gene expressions to identify candidate cancer driver genes [9]. Integrative analysis shows that CNVs strongly positively regulate gene expressions and promoter DNA methylations are weakly negatively correlated with gene expressions (Fig. 2a). Positive correlation identified 861 copy number candidate driver genes (Spearman’s correlation > 0.4 for at least one promoter probe, one-side *p*-value < 0.05), which are enriched in cell cycle (*p*-value 4.3e-04, using DAVID v6.8 [33]) and DNA repair (1.8e-03). And, negative correlation identified 223 methylation candidate drivers (correlation < − 0.4 for at least one promoter probe), which are associated with inflammatory response (2.5e-04), cell differentiation (2.5e-03), and coagulation (9.2e-03) (Additional file 7: Table S6). The second-order correlation shows that copy number candidate drivers are almost independent with methylation candidate drivers (correlation 6.3e-03, *p*-value > 0.5) (Fig. 2b). Only twenty genes are both copy number and methylation candidate drivers (Fisher’s exact test *p*-value 0.897). These results suggest that CNVs and promoter DNA methylation alterations contribute to different oncogenic processes. CNVs tend to affect basic cellular processes, and promoter DNA methylation alterations are more likely to disturb cellular responses to microenvironment.

Chromosome arms 5q, 7q, and 13q are the top three regions significantly enriched with copy number candidate drivers adjusted by gene numbers (binomial test *p*-value 6.39e-05, 1.48e-04, and 6.45-e04, respectively), and 1q and 19p are depleted (3.60e-17 and 4.66e-04). For methylation candidate drivers, 4p, 3q, and 3p are enriched (2.19e-02, 2.32e-02, and 4.85e-02) and 5q, 17q, and 12q are depleted (2.41e-02, 2.76e-02, and 4.88e-02). Region 11q13 is a hotspot of candidate drivers, with 28 copy number candiate drivers and 2 methylation candidate drivers. *CCND1* and *SERPINH1* are both copy number and methylation candidate drivers (Fig. 2c). *CCND1* is a widely studied oncogene in HCC [25]. We interestingly observed that *CCND1* is down-regulated in primary HCCs in many independent cohorts although its expression is strongly positively correlated with CNV and negatively correlated with its promoter DNA methylation. *SERPINH1*, a serpin peptidase inhibitor also named heat shock protein 47, has been reported to driver cancer cell invasion by regulating extracellular matrix gene network [34]. But, its cellular function in HCC remains unknown. Overall, this integrative analysis provides important information for studying the molecular mechanism of the metastatic HCCs.

### Recurrently differentially expressed genes between paired primary tumors and PVTTs

**Fig. 3:**
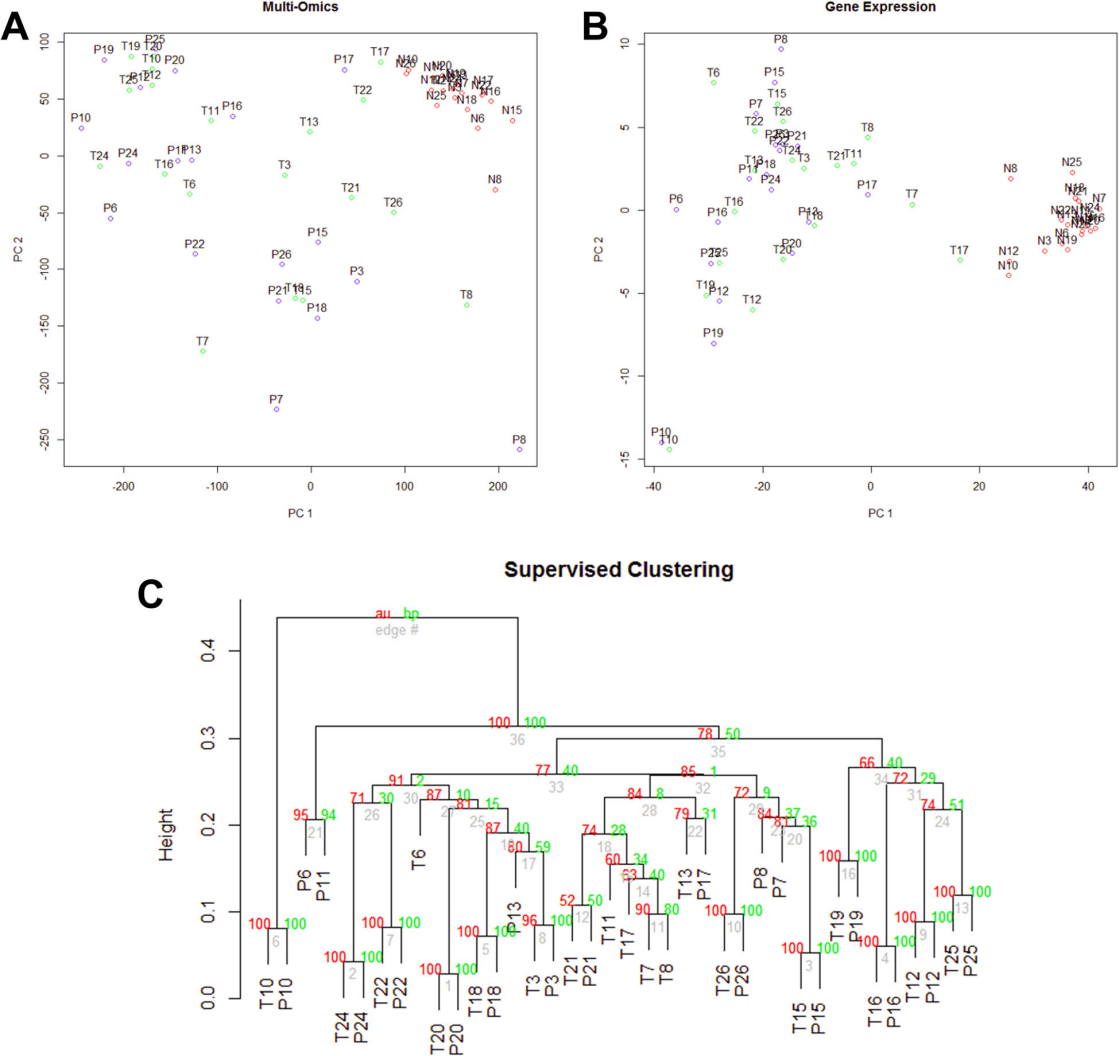
Clustering analysis reveals individualized molecular profiles between primary tumors and PVTTs. **a** The generalized principal component analysis (PCA) of multi-omics data in all the samples by LRAcluster. **b** The generalized PCA of RNA-Seq data in all the samples. **c** Supervised clustering analysis of RNA-Seq data in primary tumors and PVTTs. The top differentially expressed genes (777 genes with *p*-value < 0.05 detected by EdgeR) between primary tumors and PVTTs are used for the clustering

Another important question is whether there exist progressive molecular alterations between adjacent normal tissues, primary tumors, and PVTTs. Integrative analysis of multi-omics data shows that cancerous tissues (including primary tumors and PVTTs) are significantly different with adjacent normal tissues, and the variations between cancerous tissues are much larger than those in adjacent normal tissues (Fig. 3a). Gene expressions show similar but stronger patterns: the first component (x-axis in Fig. 3b) can accurately discriminate adjacent and cancerous tissues. To further explore the possible differences between primary tumors and PVTTs, we performed clustering by only using the differentially expressed genes between primary tumors and PVTTs (EdgeR paired test, 777 genes with raw *p*-value < 0.05). Unexpectedly, the primary tumors and PVTTs are still clustered dominantly according to their patient indexes (13 out of 19 patients) (Fig. 3c). To estimate the level of intra-patient heterogeneity between matched samples, we used the differences among adjacent normal samples as a reference: the average pairwise difference (measured as 1 - Spearman’s correlation of gene expression) is 0.019. The average pairwise difference between matched adjacent normal and primary tumors is significantly higher (difference = 0.059, Wilcox test *p*-value 7.47e-13). While, the average difference between matched primary tumors and PVTTs is comparable to the reference (difference = 0.021, *p*-value 0.167). These results suggest that the matched primary tumors and PVTTs derived from different patients have distinct progression paths, and the intra-patient tumor heterogeneity is comparable to the inter-patient variation of adjacent normal tissues.

**Fig. 4:**
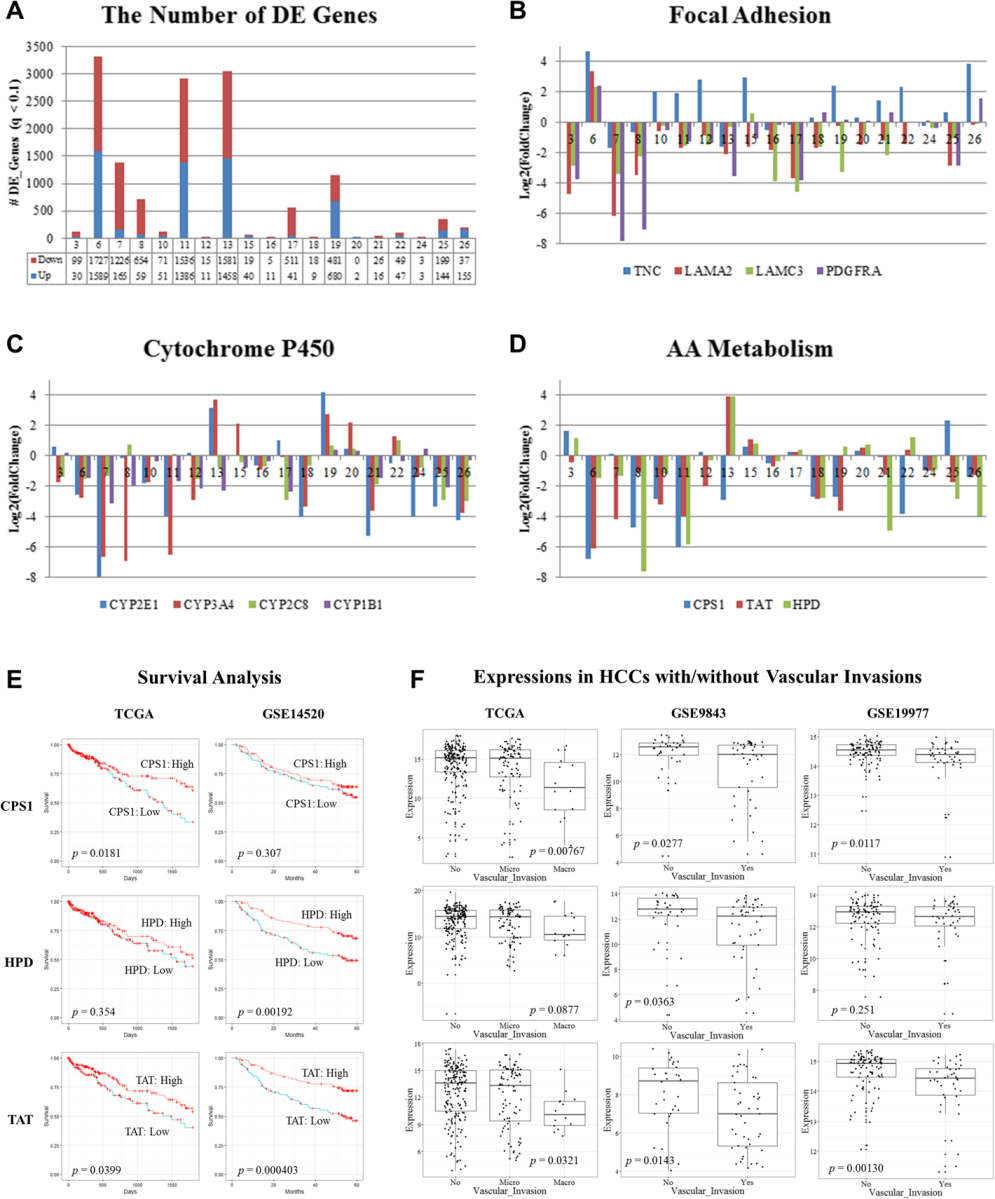
The individualized differential expression patterns between primary tumors and PVTTs identified by IDASeq. **a** The number of differentially expressed genes in each patient (q-value < 0.1). The log2 fold changes of recurrently differentially expressed genes in **b**) focal adhesion, **c** cytochrome P450 family, and **d**) amino acids metabolism. **e** Survival analyses based on *CPS1*, *HPD*, and *TAT* expressions. High (red) and low (blue) expression groups are split by median expressions. **f** Differential expression analyses of *CPS1*, *HPD*, and *TAT* between vascular invasion and non-invasion patients. The invasion group is further divided as micro-vascular invasion (second column) and macro-vascular invasion (third column) in TCGA dataset

Based on above observations, we proposed an individualized differential analysis for sequencing data (IDASeq) to identify differentially expressed genes from each pair of matched primary tumor and PVTT based on the variations estimated from all adjacent normal tissues (see Methods in Experimental Procedures). IDASeq identified different sizes of differentially expressed genes for different patients (Fig. 4a and Additional file 8: Table S7). The top three patients have ~ 3000 differentially expressed genes. But, eight patients have less than 100 differentially expressed genes. Similar results are observed for lncRNAs (Additional file 2: Figure S1 and S2).

Twenty genes were consistently differentially expressed in at least seven patients (FDR < 0.001), including *TNC*, *LAMA2*, *LAMC3*, *PDGFRA* of focal adhesion, *CYP2E1*, *CYP3A4*, *CYP2C8*, *CYP1B1* of cytochrome P450 family, and *CPS1*, *TAT, HPD* of amino acid (AA) metabolism (Table 1). The differential expressions patterns are varied in different patients (Fig. 4b-d). In the third-party cohorts, 13 genes’ expressions are strongly correlated with vascular invasion states in at least one cohort (ANOVA test, *p*-value < 0.05), in which *TAT*, *CPS1*, *CYP3A4*, and *CYP2C8* are significant in all the three cohorts. Low expressions of five genes, *CYP2E1*, *CYP3A4*, *TAT*, *CPS1*, and *HPD* are strongly associated with poor prognosis in at least one of the two cohorts with overall survival data (KM-test based on expression medians, *p*-value < 0.05). The genes of cytochrome P450 have been widely studied in hepatocellular carcinoma and many other cancer types [35–38], and *CYP2C8* is a relatively novel candidate in hepatocellular carcinoma. Cell adhesion molecules play important roles in metastasis. *LAMA2*, encoding a subunit of laminin protein, has been identified as a tumor suppressor in a recent genomic study [39] and *LAMC3* is another member of this gene family. A recent study reported that the up-regulation of *TNC* is a poor prognostic marker and can promote cancer cell migration [40]. AA metabolism is one fundamental physiological function of liver. The protein encoded by *CPS1* is the key rate-limiting enzyme of urea cycle, which is important for removing excess amino groups from cells. The proteins encoded by *TAT* and *HPD* are two enzymes of tyrosine metabolism. One study reported that *TAT* located in chr16q22 deletion region is a tumor suppressor in HCC [41]. All the three genes are significantly associated with survivals and vascular invasions (Fig. 4e and f). The altered activities of enzymes in AA metabolism pathways are usually regarded as the indication of liver function abnormality accompanied by tumor development, but only a few studies concern their roles in regulating HCC metastasis.

**Table 1:** The recurrently differentially expressed genes between matched primary tumors and PVTTs

Gene	Function	PvT	Freq	FDR	EdgeR	OS	VI
*DCN*		Down	11	< 1e-05	No	0	1
*CYP2E1*	Xenobiotics metabolism	Down	8	8.75E-05	No	1	1
*LUM*		Down	8	8.75E-05	No	0	1
*TNC*	Focal adhesion	Up	8	< 1e-05	No	0	0
*TAT*	AA metabolism	Down	8	8.75E-05	No	2	3
*LAMA2*	Focal adhesion	Down	8	8.75E-05	Yes	0	1
*SFRP4*		Down	8	8.75E-05	No	0	0
*CPS1*	AA metabolism	Down	8	8.75E-05	No	1	3
*CYP3A4*	Xenobiotics metabolism	Down	8	8.75E-05	No	1	3
*IGJ*		Down	7	1.44E-03	No	0	1
*CYP2C8*	Xenobiotics metabolism	Down	7	1.44E-03	No	0	3
*CYP1B1*	Xenobiotics metabolism	Down	7	1.44E-03	Yes	0	0
*COLEC11*		Down	7	1.44E-03	No	0	1
*IGLL5*		Down	7	1.44E-03	Yes	0	0
*ASPN*		Down	7	1.44E-03	No	0	0
*PDGFRA*	Focal adhesion	Down	7	1.44E-03	No	0	1
*ACTG2*		Up	7	3.00E-04	Yes	0	0
*INHBA*		Down	7	1.44E-03	No	0	0
*HPD*	AA metabolism	Down	7	1.44E-03	No	1	1
*LAMC3*	Focal adhesion	Down	7	1.44E-03	Yes	0	1

“PvT” denotes the direction of the differential expressions by comparing PVTTs to primary tumors; “Freq” denotes the number of patients with differentially expressed genes; “FDR” denotes the one-sided FDR calculated by permutation test; “EdgeR” denotes whether the gene is detected by EdgeR differential analysis (paired test, q-value < 0.1); “OS” denotes the number of datasets (two cohorts in total) in which the gene is significantly associated with overall survivals (KM test, *p*-value < 0.05); and, “VI” denotes the number of datasets (three cohorts in total) in which the gene is significantly associated with vascular invasion (ANOVA test, *p*-value < 0.05)

**Fig. 5:**
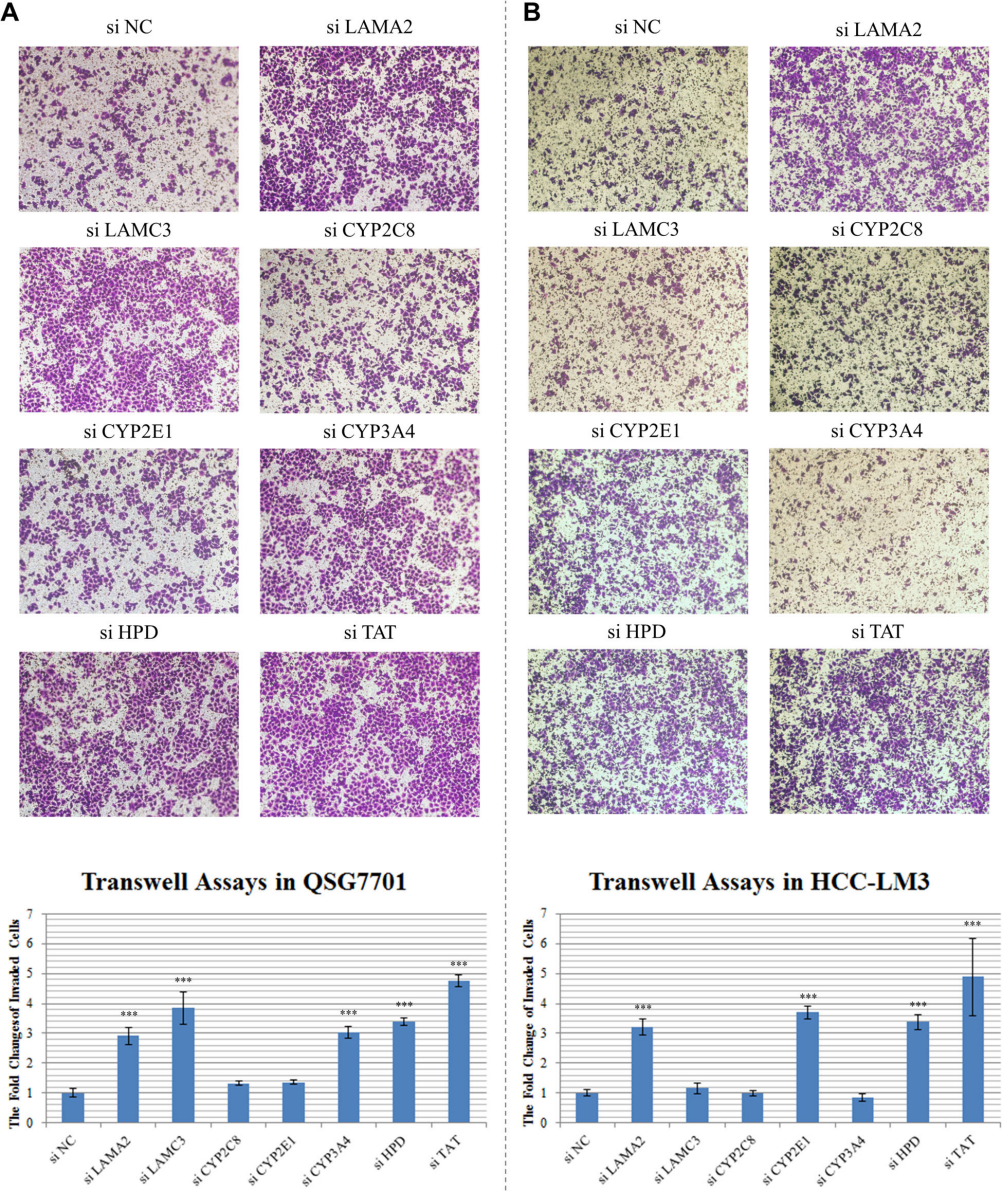
The transwell cancer cell invasion assays after siRNA knockdown of different candidate genes. **a** The number of invaded cells using QSG7701 cell line. **b** The number of invaded cells using HCC-LM3 cell line

Then, we selected seven genes for functional validations from above three categories (*LAMA2*, *LAMC3*, *CYP2C8*, *CYP2E1*, *CYP3A4*, *HPD*, and *TAT*). Results show that six out of seven genes (except *CYP2C8*) can regulate cell invasion in at least one of the two studied HCC cell lines (Fig. 5). Most interestingly, knockdown of *HPD* and *TAT*, which encode two key enzymes in in phenylalanine and tyrosine metabolism, can significantly induce cell invasions in both cell lines, which suggest that inhibition of tyrosine synthesis may cause cellular stresses and promote invasive phenotypes of cancer cells.

## Discussion

This study integratively analyzed the recurrent molecular alterations by profiling the genomic, epigenomic, and transcriptomic features of HCCs with PVTTs. Compared with several general HCC cohorts from the previous studies, few distinctive alterations were identified for these metastatic HCCs. But the alteration levels are usually more severe, such as the arm-level CNVs and chr11q13.3 focal amplification. These results suggest that these CNVs drive the progression of HCCs and the genes in the progressively over-amplified regions can be used to identify novel therapeutic targets or biomarkers. For example, the pathways of *FGF4* and *FGF19* in chr11q13.3 have already been studied as potential drug targets [42, 43].

As the previous studies, few differentially expressed genes between PVTTs and primary tumors can be found by traditional differential analysis methods which take all the studied patients as a group. We proposed a novel method IDASeq to analyze the differentially expressed genes for each patient. The main idea of IDASeq is to pool all the adjacent normal tissues to estimate the biological variances for a give expression level. To reduce the risk of over-estimation, a global permutation strategy was implemented to calculate the false discovery rate of the consistently differentially expressed genes. The IDASeq results suggest that the cancerous tissues (primary tumors and matched PVTTs) derived from different patients have highly individualized progression paths and different patients have very different levels of progressive alterations between matched primary tumors and PVTTs (range from more than 3000 to less than 10 differentially expressed genes). These results indicate that PVTT formation may have different mechanisms. For the patients with few progressively differentially expressed genes, PVTTs may form by the accumulation of randomly fallen cancer cells from the primary tumors. And, for the patients with evidential progressive alterations, PVTTs may form by highly invasive sub-clones or the randomly fallen cancer cells acquire adaptive changes for the portal vein microenvironment. Futher studies are needed to clarify these inferences. Generally, the progressive molecular alterions are much less than inter-tumor heterogeneities. Similar results are also found in previous genomic and transcriptomic studies [3, 4].

Out of twenty recurrently differentially expressed genes between matched primary tumors and PVTTs, three genes (*CPS1*, *TAT*, *HPD*) encode key enzymes of amino acid metabolism. All the three genes are significantly associated with overall survivals and vascular invasions. It should be noted that these associations may depend on a few clinical factors such as clinical stages and different treatments. Cellular assays validated that they can regulate metastatic phenotypes of HCC cells. Previously, the abnormality of AA metabolism enzymes is a key feature of liver function failure. Also, the down-regulations of these liver-specific enzymes are generally regarded as “passenger” changes along with the de-differentiated state of tumor cells. Our study established the links between these enzymes and HCC metastasis.

## Conclusions

This study identified many recurrent CNVs, abnormal DNA methylation, and differential gene expressions of metastatic HCCs with PVTTs. Integrative analysis shows that CNVs mainly regulate the genes with basic cellular functions, and promoter DNA methylations tend to mediate cellular responses to microenvironment. Individualized differential expression analysis finds that a few paitients acquire evidential progressive alterations of gene expressions between primary tumors and PVTTs. Twenty recurrently and progressively differentially expressed genes are identified. They are strongly associated with focal adhesion, xenobiotics metabolism by cytochrome P450, and amino acid metabolism, and many of them can regulate invasive phenotypes of liver-derived cell lines.

## Supplementary information

**Additional file 1:** **Table S1.** Clinical features of HCC patients.

**Additional file 2: ** Supplementary Methods and Materials. **Figure S1.** The individualized differential expression patterns of lncRNAs between primary tumors and PVTTs identified by IDASeq (q-value < 0.1). **Figure S2.** The Log2-transformed fold changes of recurrently altered lncRNAs between matched PVTTs and primary tumors. The lncRNAs are annotated by NONCODE database.

**Additional file 3:** **Table S2.** siRNA design.

**Additional file 4:** **Table S3.** CNV analysis.

**Additional file 5:** **Table S4.** DNA methylation analysis.

**Additional file 6:** **Table S5.** RNA sequencing analysis.

**Additional file 7:** **Table S6.** Integrative analysis with RNA-seq.

**Additional file 8:** **Table S7.** IDASeq analysis.

## Data Availability

All the data can be accessed via NCBI GEO SuperSeries GSE77276 (GSE77275 for CNVs and SNPs, GSE77269 for DNA methylations, GSE76903 for miRNA-seq and GSE77509 for RNA-seq) (https://www.ncbi.nlm.nih.gov/geo/query/acc.cgi?acc=GSE77276).

## Abbrevations

AA: Amino acids
CIMP: CpG island methylator phenotype
CNV: Copy number variation
GEO: Gene Expression Omnibus
HCC: Hepatocellular carcinoma
PVTT: Portal vein tumor thrombus
TCGA: The Cancer Genome Atlas
