# Supplementary material for: Integrative molecular analysis of metastatic hepatocellular carcinoma
Source: BMC Med Genomics. 2019 Nov 13;12:164. doi: 10.1186/s12920-019-0586-4 (PMC6854708; doi:10.1186/s12920-019-0586-4)
Supplement: Supplementary file 1 — Additional file 1: Table S1. Clinical features of HCC patients. [file 12920_2019_586_MOESM1_ESM.docx]

**Table S1**. Clinical features of HCC patients.

| **Variable** | **Statistics** |
| --- | --- |
| **Age** (median, range)  ≤ 50  > 50 | 49, [35-66]  10 (52.6%)  9 (47.4%) |
| **Gender**  Male  Female | 17 (89.5%)  2 (10.5%) |
| **Etiology**  HBV+  HCV+  Cirrhosis | 17 (89.5%)  0 (0%)  16 (84.2%) |
| **Tumor size** (median, range)  ≤ 5cm  > 5cm | 8, [2.4-38]  3 (15.8%)  15 (78.9%) |
| **AFP before surgery**  ≤ 200  > 200 | 6 (31.6%)  13 (68.4%) |
| **Tumor numbers**  Single  Multiple | 15 (78.9%)  2 (10.8%) |
| **Edmondson-Steiner grade**  3  4 | 17 (89.4%)  1 (5.3%) |
