# Supplementary material for: Integrative molecular analysis of metastatic hepatocellular carcinoma
Source: BMC Med Genomics. 2019 Nov 13;12:164. doi: 10.1186/s12920-019-0586-4 (PMC6854708; doi:10.1186/s12920-019-0586-4)
Supplement: Supplementary file 2 — Additional file 2: Supplementary Methods and Materials. Figure S1. The individualized differential expression patterns of lncRNAs between primary tumors and PVTTs identified by IDASeq (q-value < 0.1). Figure S2. The Log2-transformed fold changes of recurrently altered lncRNAs between matched PVTTs and primary tumors. The lncRNAs are annotated by NONCODE database. [file 12920_2019_586_MOESM2_ESM.docx]

# Supplementary Methods and Materials

## Methods: individualized differential analysis for sequencing data (IDASeq)

IDASeq was implemented to identify the differentially expressed transcriptional units (protein-coding genes or lncRNAs) for each individual using paired sequencing data. In this study, paired samples of adjacent normal tissues, primary tumors and portal vein thrombi were used for RNA-Seq. For count-based RNA-Seq expression data, it is commonly assumed that the variances conditional on given expression mean $\sigma^{2}|\mu$ fit the probabilistic model of negative binomial distribution $\sigma^{2}|\mu=\mu+\alpha\mu^{2}$ (Anders and Huber, 2010; Love *et al*., 2014). To identify the significantly differentially expressed transcripts for each individual, the major challenge is to estimate the variances $\sigma^{2}|\mu$ from the paired sequencing data without biological replicates (Wang *et al*., 2010; Feng *et al*., 2012). Our analyses show that the adjacent normal tissues are closely clustered. From biological view, IDASeq assumed that the gene expression data of the adjacent normal tissues can be used as biological meaningful replicates to estimate the variances $\sigma^{2}|\mu$. Then, the statistical significance of the expression difference for gene *i* between *j*-th pair of primary tumor and portal vein thrombus $d_{ij}=e_{i,j}^{p}-e_{i,j}^{t}$ can be assessed by $z_{ij}=\frac{d_{ij}}{\sqrt{{2\sigma^{2}|}_{\mu_{ij}}}}$ where $\mu_{ij}=\frac{e_{i,j}^{p}+e_{i,j}^{t}}{2}$ ($z_{ij}$ follows standard normal distribution).

The detailed steps of IDASeq:

1) Calculate the expression mean of gene *i* in adjacent normal tissue: $\mu_{i}^{n}$;

2) Fit the variances of gene *i* using negative binomial distribution: ${\left( \sigma_{i}^{n} \right)^{2}|}_{\mu_{i}^{n}}$;

3) Calculate the expression difference $d_{ij}=e_{i,j}^{p}-e_{i,j}^{t}$ and mean $\mu_{ij}=\frac{e_{i,j}^{p}+e_{i,j}^{t}}{2}$ of gene *i* in *j*-th paired primary tumor and portal vein thrombus;

4) Use the variances of genes in adjacent normal tissues with similar expression mean to estimate the variance of gene *i* in *j*-th paired samples: ${\sigma^{2}|}_{\mu_{ij}}$. In this study, we used the median of top 100 genes most similar genes to do the estimation. Compared with local regression such as LOWESS or spline regression, median smoothing can reduce the effect of outliers during local variance estimation;

5) Calculate $z_{ij}=\frac{d_{ij}}{\sqrt{{2\sigma^{2}|}_{\mu_{ij}}}}$ and the corresponding p-value based on standard normal distribution (adjusted by BH-correction).

In this study, the differentially expressed genes were detected with adjusted p-value < 0.1 for each patient.

To estimate the statistical significances of the recurrently differentially expressed genes, a permutation test was used: 1) randomly sample size-matched genes according to the number of differentially expressed genes for each patient (the directions of differential expressions are also matched); 2) calculate the numbers of gene recurrences in all patients; 3) repeat the sampling processes *N* times (10,000 in this study) to estimate the null distribution of the recurrences; and 4) calculate FDRs for different levels of recurrences (up-regulated and down-regulated are calculated separately).

**References**

Anders S and Huber W. Differential expression analysis for sequence count data. *Genome Biol* 2010, 11(10):R106.

Feng J, Meyer CA, Wang Q, Liu JS, Liu SL, Zhang Y. GFOLD: a generalized fold change for ranking differentially expressed genes from RNA-seq data. *Bioinformatics* 2012, 28(21):2782-2788.

Love MI, Huber W and Anders S. Moderated estimation of fold change and dispersion for RNA-seq data with DESeq2. *Genome Biol* 2014, 15(12):550.

Wang L, Feng Z, Wang X, Wang X and Zhang X. DEGseq: an R package for identifying differentially expressed genes from RNA-seq data. *Bioinformatics* 2010, 26(1):136-138.

## Supplementary Figure S1


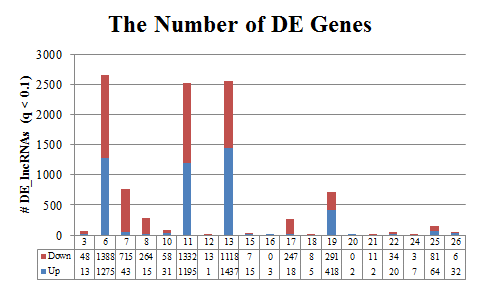


**Figure S1**. The individualized differential expression patterns of lncRNAs between primary tumors and PVTTs identified by IDASeq (q-value < 0.1).

## Supplementary Figure S2


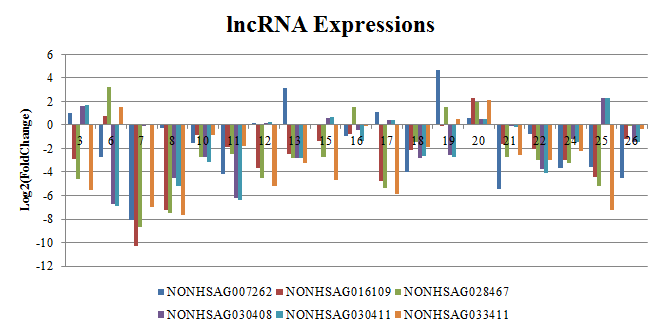


**Figure S2**. The Log2-transformed fold changes of recurrently altered lncRNAs between matched PVTTs and primary tumors. The lncRNAs are annotated by NONCODE database.
